# Modeling the effect of boost timing in murine irradiated sporozoite prime-boost vaccines

**DOI:** 10.1371/journal.pone.0190940

**Published:** 2018-01-12

**Authors:** Cristina Fernandez-Arias, Clemente F. Arias, Min Zhang, Miguel A. Herrero, Francisco J. Acosta, Moriya Tsuji

**Affiliations:** 1 HIV and Malaria Vaccine Program, Aaron Diamond AIDS Research Center, Affiliate of The Rockefeller University, New York, NY, United States of America; 2 Grupo Interdisciplinar de Sistemas Complejos (GISC), Madrid, Spain; 3 Departamento de Matemática Aplicada, Universidad Complutense de Madrid, Madrid, Spain; 4 Department of Pathology, University of New York, NY, United States of America; 5 Departamento de Ecología, Universidad Complutense de Madrid, Madrid, Spain; Institut de recherche pour le developpement, FRANCE

## Abstract

Vaccination with radiation-attenuated sporozoites has been shown to induce CD8+ T cell-mediated protection against pre-erythrocytic stages of malaria. Empirical evidence suggests that successive inoculations often improve the efficacy of this type of vaccines. An initial dose (prime) triggers a specific cellular response, and subsequent inoculations (boost) amplify this response to create a robust CD8+ T cell memory. In this work we propose a model to analyze the effect of T cell dynamics on the performance of prime-boost vaccines. This model suggests that boost doses and timings should be selected according to the T cell response elicited by priming. Specifically, boosting during late stages of clonal contraction would maximize T cell memory production for vaccines using lower doses of irradiated sporozoites. In contrast, single-dose inoculations would be indicated for higher vaccine doses. Experimental data have been obtained that support theoretical predictions of the model.

## Introduction

Malaria is a severe disease that ranks among the most prevalent infections in tropical areas throughout the world. Approximately 250-300 million people become infected yearly with relatively high rates of morbidity and mortality [1]. The widespread occurrence and the increasing incidence of malaria in many countries underscore the need for developing new methods of controlling this disease, which includes more effective vaccines. Most vaccine efforts are directed against the pre-erythocytic stages (sporozoites and liver stages), and blood stages [2]. The finding that vaccination with radiation-attenuated sporozoites can induce temporary protection, i.e. sterile immunity, against malaria infection not only in experimental animals, but also in humans [3–6], demonstrated the feasibility of effective vaccination against this disease.

Experimental studies have shown that protective immunity against pre-erythrocytic stages is mediated in part by T cells, particularly CD8+ T cells [7, 8]. For instance, in vivo depletion of CD8+ T cells abrogated sporozoite-induced protective immunity in mice [9, 10]. Moreover, the adoptive transfer of CD8+ T cell clones specific for sporozoite antigens was shown to confer protection against sporozoite challenge in naïve mice [11, 12]. More recently, it has been observed in transgenic mice expressing a T cell receptor (TCR) recognizing the *Plasmodium* SYVPSAEQI epitope that transgenic CD8+ T cells mediate protection against malaria [13]. Finally, it has also been shown that immunizing with recombinant adenovirus expressing the *Plasmodium yoelli* circumsporozoite protein (CSP) could induce a potent protective anti-malarial immunity, which was mediated by CD8+ T cells [14]. To date, several vectors have been shown to increase CD8+ T cell protection, including recombinant adenovirus expressing the *Plasmodium yoelli* circumsporozoite protein (CSP) [14, 15], DNA vaccines [16, 17], recombinant protein vaccines [18], or viral vector vaccines [19].

Empirical evidence suggests that prime-boost (PB) regimes can improve the efficacy of this type of vaccines, as compared to single-dose strategies [11, 20, 21]. In PB vaccines, an initial inoculation (prime) serves to generate a population of antigen-specific effector T cells, and subsequent inoculations of the same or a different vector (boost) promote expansion of this population, thus increasing the pool of long-lasting specific immune memory [22]. The rationale of PB strategies can therefore be viewed as forcing T cell population dynamics so as to maximize the production of memory T cells, thus ensuring a sustained protection against future challenges [23, 24].

**Fig 1:**
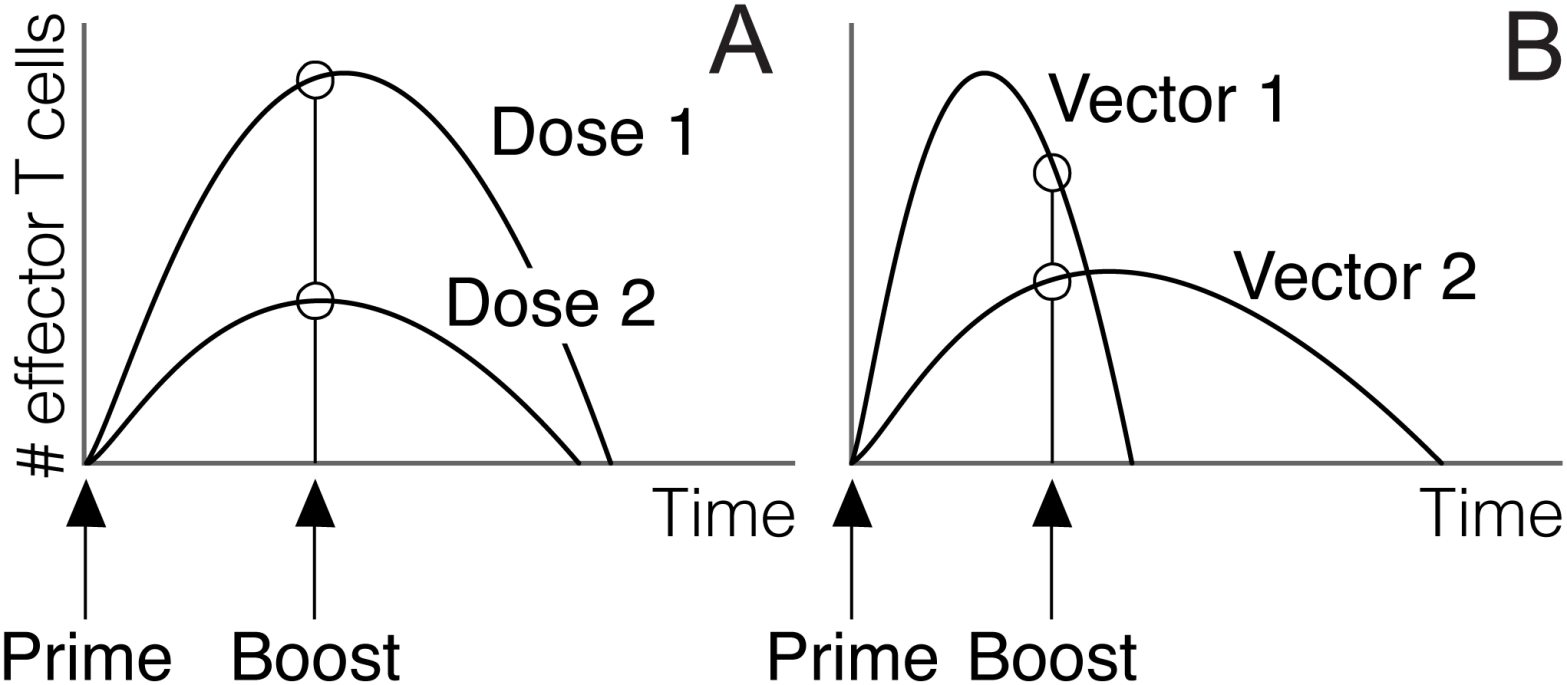
The effect of boosting at a fixed time after priming is expected to depend on T cell dynamics triggered by priming. A) Different antigen doses elicit T cell responses that can differ in their magnitude and duration [24, 31–36]. In this context, boost antigens inoculated at equal intervals after priming will interact with populations of effector T cells that differ in size. B) T cell populations primed with different vectors can be at different stages of the response (clonal expansion vs. clonal contraction) at a given time after the first injection.

In spite of the central role played by T cell population dynamics in the performance of PB vaccines, dynamic aspects of T cell responses are often neglected when designing vaccination protocols against malaria. For instance, the timing of booster immunizations is often described in vaccine specifications in terms of days or weeks after priming [25–30], ignoring the fact that boosting takes place in the context of an initial T cell response elicited by priming. In this regard, it is worth noting that T cell immune responses to different pathogens vary in quantitative features such as the peak of clonal expansion or the duration of the response [31–33]. Similarly, alternative vaccine vectors (or different doses of the same vector) differ in the duration and/or magnitude of clonal expansion they elicit on T cells [24, 34–36]. Therefore, the status of T cell populations at a fixed time after priming is expected to vary depending on the particular nature and dose of the agent used for priming (see Fig 1).

Bearing these facts in mind, the question naturally arises of understanding how T cell dynamics triggered by priming can be modified by boost to generate a robust immune memory against target *Plasmodium* epitopes. In order to address this issue, we will make use of population mechanics, a mathematical framework that allows to model the behavior of T cell populations during immune response [37, 38]. The organization of this article is as follows. We will begin by formulating a model of T cell clonal expansion and contraction that accounts for the main features of T cell response as described in the literature. We will then use this model to simulate the effects of PB vaccines on T cell population dynamics. Finally, we will test the predictions of this model concerning the effect of boost timing on the formation of memory T cells in mice vaccinated with irradiated *Plasmodium* sporozoites. In particular, we will consider the effect of PB regimes on liver-resident memory T cells, a phenotypically differentiated subset of T cells [39] responsible for observed immune protection after vaccination with irradiated sporozoites [40].

## Results

### Dynamics of effector and memory T cells immune response

In previous works we have used population mechanics to model the behavior of effector T cells during immune response [38], and that of naïve and memory T cells in homeostasis [37] (see also S1 File). We argued there that T cell populations show inertia and elasticity, features that admit a straightforward formulation in terms of simple second order differential equations. From this approach, stimuli that foster T cell proliferation can be understood as forces acting on T cell populations. For instance, clonal expansion of effector T cells during immune response can be viewed as driven by an antigenic force. As for naïve and memory T cells in homeostasis, such external force is provided by interleukins (ILs), specifically by IL-7 and IL-15, and also by antigenic stimulation provided by antigen-presenting cells (APCs) in the secondary lymphoid organs [41–44].

In this section we will use this approach to model the simultaneous dynamics of effector and memory T cells during an immune response. In order to do so, we will consider they constitute two separate populations that compete for antigen stimulation. On their turn, since newly formed memory T cells are equipped with IL-7 and IL-15 receptors [45], they compete for interleukins with the population of pre-existing memory T cells. The dynamics of homeostatic ILs (that will generically be labelled as *H*) will be modeled by taking IL-7 as a reference. It has been observed that the amount of available IL-7 results from the balance between a relatively constant rate of production in lymphoid tissues, and its consumption by T cells [37, 46]. Finally, the pathogen is assumed to proliferate at a constant rate *α* and to be removed by effector T cells at rate *β*.

With all these elements, the dynamics of T cell populations during acute infections can be modeled by means of the following set of differential equations:

$$\begin{array}{c} \left\{ \begin{array}{c} E_{a}^{\prime \prime }\left(t\right)=-kE_{a}\left(t\right)+\frac{E_{a}\left(t\right)}{E_{a}\left(t\right)+M_{a}\left(t\right)}F_{A}\left(t\right)\\ M_{a}^{\prime \prime }\left(t\right)=-cM_{a}^{\prime }\left(t\right)-kM_{a}\left(t\right)+\frac{M_{a}\left(t\right)}{E_{a}\left(t\right)+M_{a}\left(t\right)}F_{A}\left(t\right)+\frac{M_{a}\left(t\right)}{M\left(t\right)+M_{a}\left(t\right)}F_{H}\left(t\right)\\ M^{\prime \prime }\left(t\right)=-cM^{\prime }\left(t\right)-kM\left(t\right)+\frac{M(t)}{M\left(t\right)+M_{a}\left(t\right)}F_{H}\left(t\right)\\ H^{\prime }\left(t\right)=\varphi -\mu M\left(t\right)-\mu M_{a}\left(t\right)\\ P^{\prime }\left(t\right)=\alpha P\left(t\right)-\beta \left(E_{a}\left(t\right)+M_{a}\left(t\right)\right)P\left(t\right), \end{array}\right. \end{array}$$ (1)

where *E*_*a*_ and *M*_*a*_ are the populations of effector and memory T cells that are activated during the immune response, *M* is the pool of memory T cells existing before the infection and *P* is the pathogen triggering the immune response. *F*_*A*_(*t*), and *F*_*H*_(*t*) are the antigenic force originated by pathogen *P* and the homeostatic force created by interleukin *H* at time *t* respectively. Parameters *k* and *c* represent the elastic constant and the damping coefficient of T cell populations. Parameter *φ* is the constant rate of interleukin *H* production and *μ* is the rate of interleukin consumption by memory T cells. Finally, parameters *α* and *β* are the pathogen rates of proliferation and clearance by T cells respectively. Eq (1) are valid for positive values of all the variables (see S1 File and Fig A therein). For the sake of simplicity we will assume that antigenic and homeostatic forces are proportional to the size of the pathogen population and to the amount of homeostatic interleukin respectively (i.e., *F*_*A*_(*t*) = *λ*_*AP*_ *P*(*t*) and *F*_*H*_(*t*) = *λ*_*H*_ *H*(*t*), for positive values of *λ*_*AP*_ and *λ*_*H*_). Greater values of parameter *λ*_*AP*_ thus represent either higher affinities of T cell receptors for their cognate antigens, or higher levels of antigen expression by the pathogen [47, 48].

**Fig 2:**
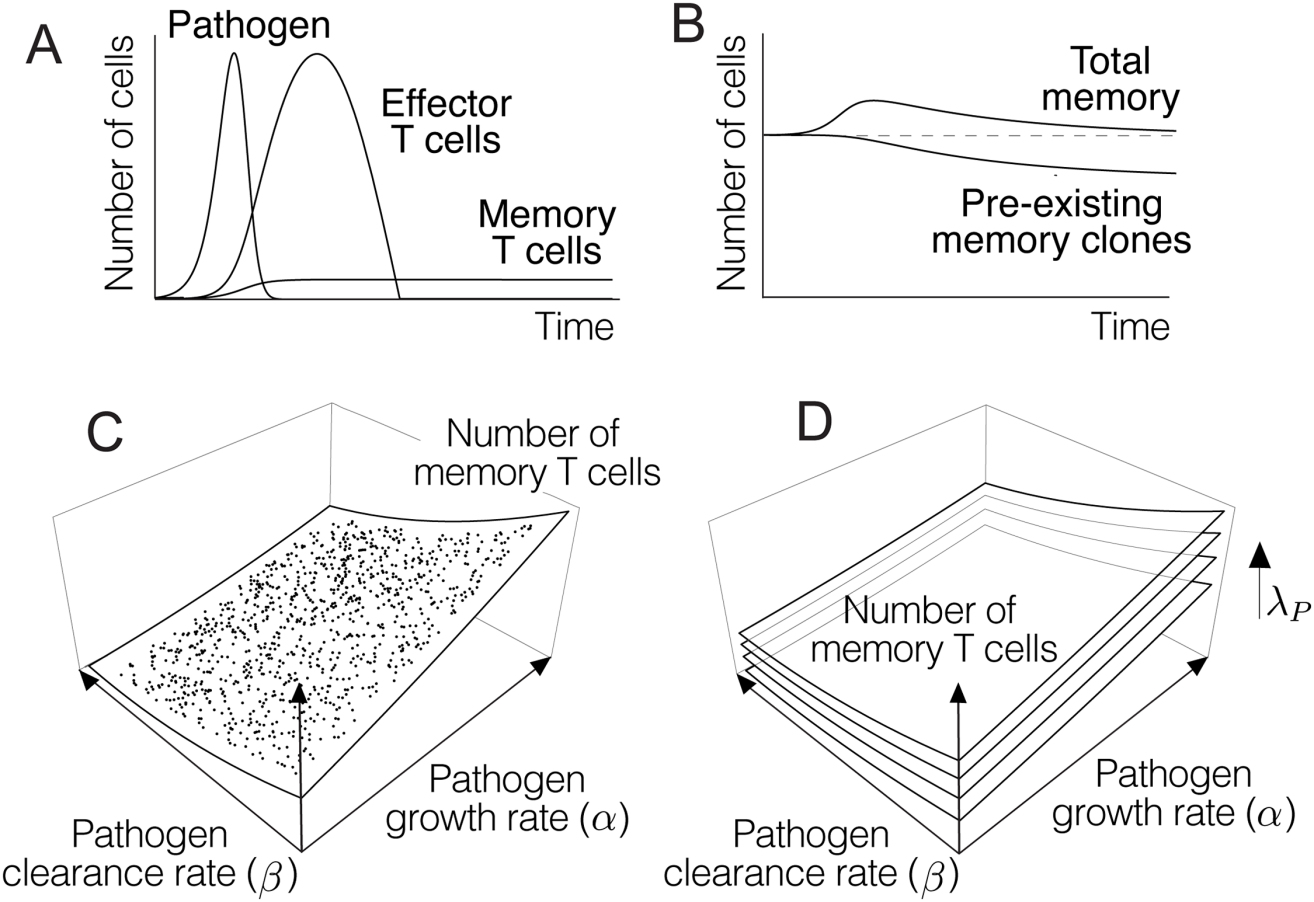
Behavior of solutions of Eq (1). A) Numerical simulations of Eq (1) reproduce the qualitative dynamics of T cell clonal expansion and contraction [32, 49]. B) In agreement with empirical data, a transient increase in total memory is eventually damped out to a structural carrying capacity (dashed line), which entails the loss of some pre-existing memory T cells [37, 52]. C) According to Eq (1), infections by pathogens with higher growth rates or lower clearance rates result in the formation of more memory T cells. D) The number of memory T cells also increases with parameter *λ*_*AP*_, i.e, clones showing higher affinities for antigens of the pathogen, or clones targeting more abundant antigens produce more memory T cells. The values of the parameters used in A and B are the following (in suitable units): *k* = 150, *c* = 40, *λ*_*P*_ = 50, *α* = 50, *β* = 0.01, *y*_0_ = 10^3^, *μ* = 1, *φ* = 10^6^, *λ*_*H*_ = 10, and *T*_*P*_(0) = 10. Each dot in C corresponds to a single numerical simulation of the model using the same parameters as in B, except for *α* and *β*, which are randomly chosen within the ranges [20, 40] and [0.5, 1.2] respectively. Parameter values in D are the same as in C, with *λ*_*AP*_ taking the values 25, 55, 85 and 115.

Fig 2 summarizes the main features of the dynamics described by numerical simulation of Eq (1). As shown there, this model captures the qualitative dynamics of clonal expansion and contraction displayed by effector T cells during an immune response (Fig 2A) (see e.g. [49–51]). As shown in Fig 2A, the system described by Eq (1) reaches a steady state in which both the pathogen and the population of effector T cells go extinct. In this steady state, if condition *kc* > *λμ* holds, the size of the memory pool is constrained by a carrying capacity *K* = *φ*/*μ* (see [37] for further details). In agreement with published data [52], the total number of memory T cells temporarily increases in acute infections (Fig 2B). However, owing to the limitations imposed by the underlying carrying capacity, the population eventually returns to equilibrium, which entails the loss of some pre-existing memory T cells (Fig 2B). Hence, each episode of infection changes the relative proportion of clones in the pool of memory T cells [37, 53].

Also in line with empirical observations, the model predicts that the extent of such changes is determined by the magnitude of the clonal expansion of T cells that respond to the pathogen (Fig 2C) [54, 55]. In turn, the peak of clonal expansion is known to be related to the affinity of effector T cells for their cognate antigens [20, 31, 56], and can be modulated by the presence of inflammatory cytokines [57]. As noted above, these situations can be modeled by assigning higher values to parameter *λ*_*AP*_ in Eq (1) (see Fig 2D). It follows that clones of T cells that respond to more threatening pathogens (i.e., pathogens displaying higher growth rates or lower clearance rates) occupy larger fractions of the immune memory. This would provide a strategy to reduce the possibility of such a clone disappearing as a consequence of the formation of new memory T cells in the course of future infections [37].

According to Eq (1), memory T cells also undergo clonal expansion but, unlike effector T cells, contraction is prevented in this case by the action of interleukin-driven homeostatic force. The observed differences in the magnitude of expansion between effector and memory T cells [50, 51] emerge in the model from the damping coefficient of the latter (*c* > 0). According to our model, the expansion of the population of memory T cells can occur through any of two alternative mechanisms. For instance, it can result from progressive differentiation of effector T cells as the infection rages [57, 58]. Alternatively, it may arise from direct proliferation of memory T cells resulting from asymmetric division of activated naïve T cells [59, 60]. We remark that the previous equations do not account for the mechanisms of memory formation. As a matter of fact, the model presented here is intended to reproduce the population-scale dynamics of T cells that will end up displaying a memory phenotype, irrespectively of the particular mechanisms involved in this process.

Parameterizing the previous equations by fitting their solutions to empirical data is a challenging issue, owing to the difficulty to obtain sufficiently accurate data about the dynamics of T cells and pathogens during an immune response. For instance, it is difficult to identify the specific clones that respond to a given antigen, or to measure the number of effector T cells produced in the course of immune responses without interfering with their normal dynamics. Moreover, T cell response depends on histocompatibility antigens, which differ among individuals of a population, so that the same set of antigens can trigger quantitatively different responses in different individuals [24, 61]. These problems obviously limit the power of our model to make precise quantitative predictions, such as the exact number of memory cells that will be formed in the course of acute infections or after inoculating a particular vaccine. However, the previous discussion suggests that Eq (1) provide a compact, simple model that captures the main qualitative features of T cell dynamics during immune response. Accordingly, we suggest that this model can provide valuable insight to be applied in the design of PB vaccines against malaria. In particular, we will show that it can be used to analyze the effect of boost timing on the formation of memory T cells, a variable that has been observed to correlate with vaccine-induced protection [20, 62, 63].

### Dynamics of effector and memory T cells in prime-boost vaccines with irradiated sporozoites

In order to use Eq (1) in the context of intravenous PB vaccines with irradiated sporozoites two considerations are in order. First, *Plasmodium* sporozoites do not divide in the host. Instead, they migrate to the liver and move through liver cells in a process termed transcytosis [64]. This behavior continues until they successively differentiate into schizonts and trophozoites, which marks the initiation of the erythrocytic stage [65]. In this process, infected liver cells end up displaying *Plasmodium* antigens, so they become targets of CD8+T cell immune response [66, 67]. As for irradiated sporozoites, they do not differentiate into trophozoites but nonetheless are able to infect liver cells and to perform transcytosis with the same efficiency as normal sporozoites [68, 69]. Therefore, transcytosis performed by irradiated sporozoites causes a progressive increase in the number of wounded of infected hepatocytes, and consequently in the amount of antigens available for CD8+ T cells stimulation.

The second issue that arises when using Eq (1) to model PB vaccines is of a general nature and concerns potential interactions between prime and boost antigens. Particularly, boost antigens can be opsonized (i.e., targeted by specific antibodies) as consequence of humoral responses triggered by prime antigens, which can affect their dynamic parameters [22, 70].

To the best of our knowledge, no quantitative data about rates of liver cell infection by irradiated sporozoites are currently available in the literature. Therefore, published evidence is insufficient to build precise models of how these rates might change between irradiated sporozoites administered in prime and boost inoculations. However, we suggest that Eq (1) can still be used to model T cell dynamics elicited by PB vaccines against malaria. In order to do so, we will assume that the number of infected cells (and consequently the amount of available antigen) first grows exponentially and then decreases by the action of activated T cells. On the other hand, we will only analyze homologous PB strategies, i.e., regimes in which irradiated sporozoites are inoculated both as prime and boost vectors. For the sake of simplicity we will model vaccine protocols consisting in two successive inoculations of irradiated sporozoties (see Fig B in S1 File). Finally, we will implement the same antigen dynamics during prime and boost by using the same growth and removal parameters (*α* and *β* respectively) in both phases of the vaccine. We remark that our model could be adapted to incorporate future information about the dynamics of cell infection by irradiated sporozoites. Even at the current stage our model can be used to understand dynamic aspects of T cell response that are not obvious at first glance, but might be taken into account in the design of PB protocols.

**Fig 3:**
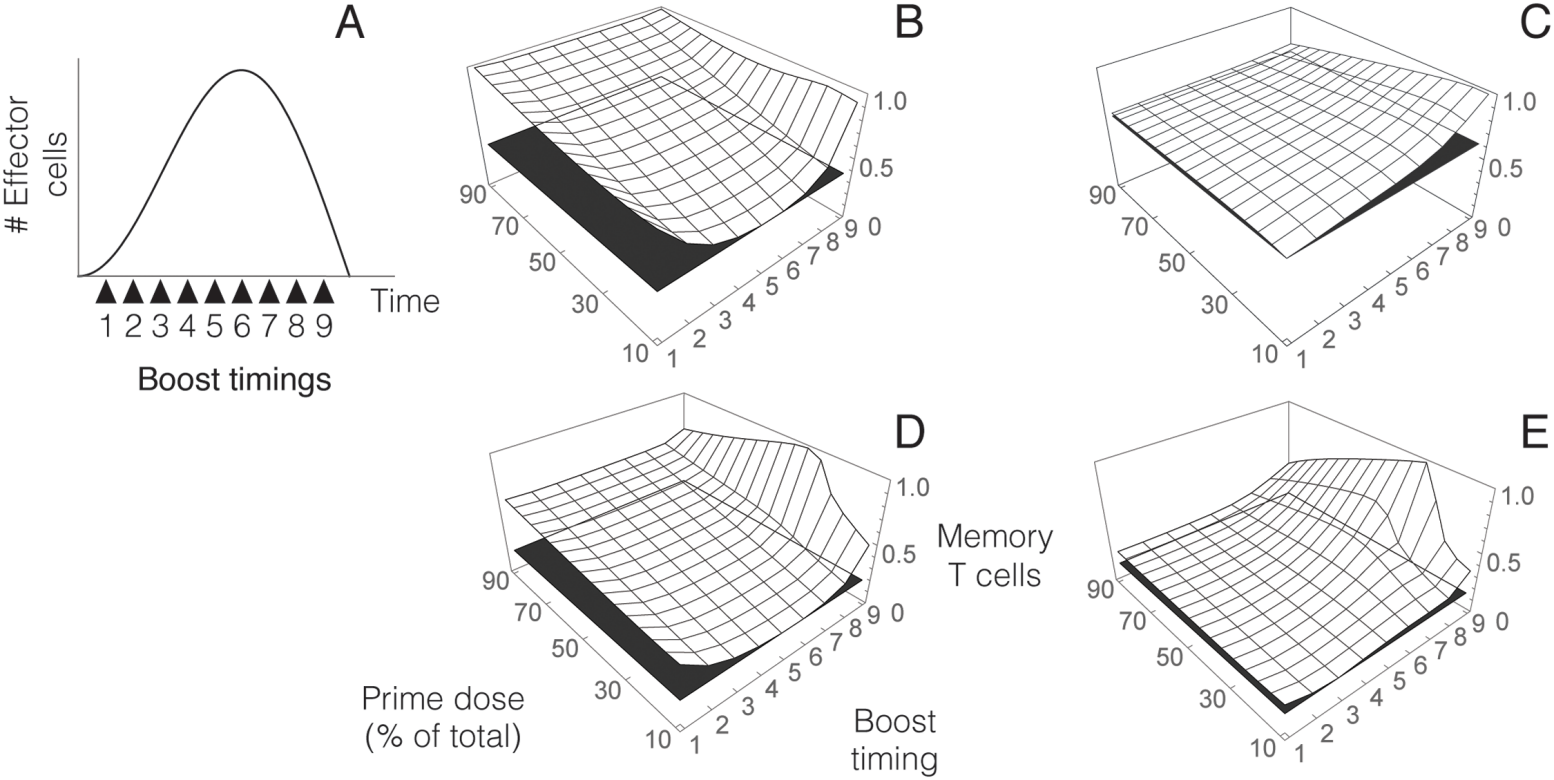
Numerical simulations of the model of homologous PB regimes with different parameters values. A). Behavior of effector T cells after prime according to Eq (1). PB protocols are defined by choosing nine boost timings equally distributed between the initiation of the T cell response and the end of clonal contraction. B) Memory T cells formed for PB protocols that differ in prime dose and boost time, using a high total antigen dose. C) As B with lower antigen load. Results are normalized relative to the maximum in each figure. Black planes show the minimum value of memory formation for comparison. D,E) Same as B and C for a different choice of parameter values. The values of the parameters used in these simulations are the following (in suitable units): (B) *A*_0_ = 20000, *λ*_*A*_ = 450, *c* = 40, *k* = 250, *λ*_*H*_ = 10, *φ* = 10^7^, *μ* = 1, *α* = 15, *β* = 0.04 and *E*_*a*_(0) = *M*_*a*_(0) = 10. (C) Same as in B, with *A*_0_ = 200. (D) *A*_0_ = 10000, *λ*_*P*_ = 250, *c* = 50, *k* = 120, *λ*_*H*_ = 10, *φ* = 10^7^, *μ* = 1, *A*_0_ = 10^7^, *μ* = 1, *α* = 2, *β* = 0.2 and *E*_*a*_(0) = *M*_*a*_(0) = 10. (E) Same as in C, with *A*_0_ = 1000.

In order to model different PB protocols we proceed as follows. First, for a given set of parameters we use Eq (1) to establish the duration of the T cell response triggered by priming with a particular antigen dose. Boost timings are then chosen between the onset of clonal expansion and the end of clonal contraction (Fig 3A). Finally, we define a boost dose and simulate the dynamics of the resulting PB regime (Fig B in S1 File). Numerical simulations of this model for different antigen doses and different parameter values show that memory formation depends on the distribution of antigen between prime and boost, as well as on boost timing (Fig 3B–3E). Moreover, optimal combinations of antigen distribution and boost timing seem to depend on the total dose of antigen (see Fig 3B–3D and 3C–3E). In the particular examples shown in Fig 3, vaccines with higher antigen load produce less memory T cells at intermediate boost timings and with low boost doses (Fig 3B and 3C)). On the other hand, for lower antigen doses combinations of late boost times and low or intermediate prime doses result in higher memory production (Fig 3D and 3E).

### Modeling the effect of boost timing on memory T cell formation

The results presented in the previous section correspond to four particular choices of model parameters. They suggest that relative performance (in terms of memory formation) of PB protocols that differ in boost doses and timings might obey to distinct patterns related to total antigen doses. In this section we will show that this behavior is consistent across a wide range of parameter values.

**Fig 4:**
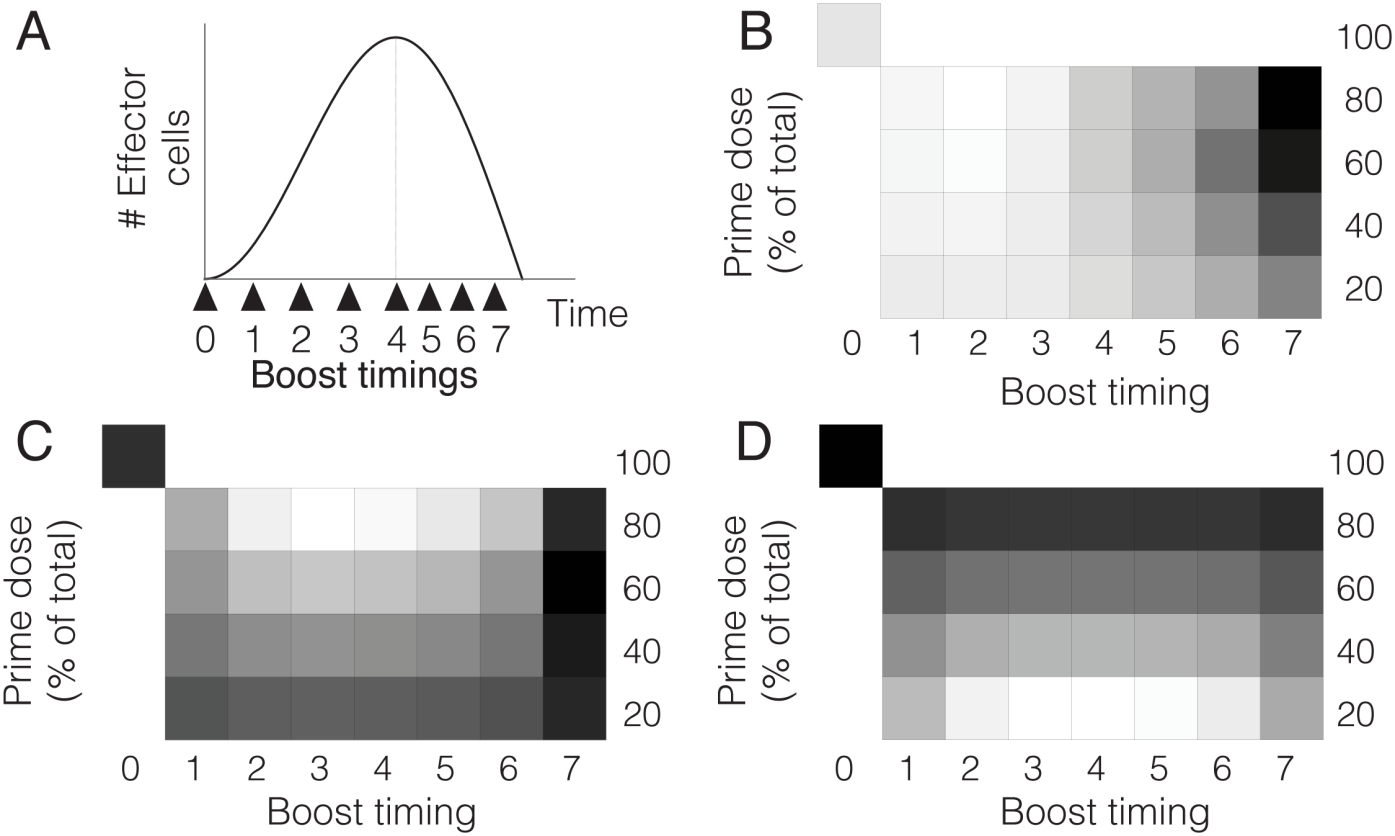
Numerical simulations of the model of T cell dynamics in PB vaccines. Vaccination protocols are defined by initially setting a total antigen dose. A percentage of this total dose (20, 40, 60 or 80%) is used for priming. A) Dynamics of effector T cells (as described by Eq (1)) triggered by priming. The remaining antigen is inoculated at different boost timings chosen relative to clonal expansion and contraction (black arrows). The point marked as zero corresponds to administering all the antigen dose in a single inoculation. B,C,D) Relative performance of PB protocols for low, intermediate and high antigen doses (*A*_0_ = 2 × 10^2^, *A*_0_ = 2 × 10^3^ and *A*_0_ = 2 × 10^4^) respectively (see Fig A in S1 File). Darker colors indicate greater memory production. These results correspond to 5000 numerical simulations of the model for the indicated antigen doses, with the rest of the model parameters (in suitable units) randomly chosen within the following intervals: 50 ≤ *λ*_*A*_ ≤ 500, 20 ≤ *c* ≤ 80, 100 ≤ *k* ≤ 200, 5 ≤ *λ*_*H*_ ≤ 20, 10^5^ ≤ *φ* ≤ 10^7^, 1 ≤ *μ* ≤ 10, 5 ≤ *α* ≤ 15, 0.001 ≤ *β* ≤ 0.5 and 1 ≤ *E*_*a*_(0) = *M*_*a*_(0)≤10.

Fig 4 displays the results of numerical simulations of the model for PB protocols in which a fixed amount of antigen load is delivered in different prime and boost doses, and at different boost times. Specifically, for a given dose of antigen we define several vaccination scenarios that differ in the distribution of such dose between prime and boost. Boost timings are then selected for each scenario according to clonal expansion and contraction resulting from the prime dose delivered (see Fig 4A).

Numerical simulations displayed in Fig 4 confirm and generalize the trends described in the previous section. In particular, for lower total antigen loads, memory T cell production tends to be greater for low boost doses, inoculated at later stages of clonal contraction (see Fig 4B). If total antigen load is increased, then maximum memory T cell production corresponds to lower prime doses (Fig 4C). Finally, further increasing total antigen load shifts this maximum towards single inoculation vaccines or PB regimes with higher prime doses.

**Fig 5:**
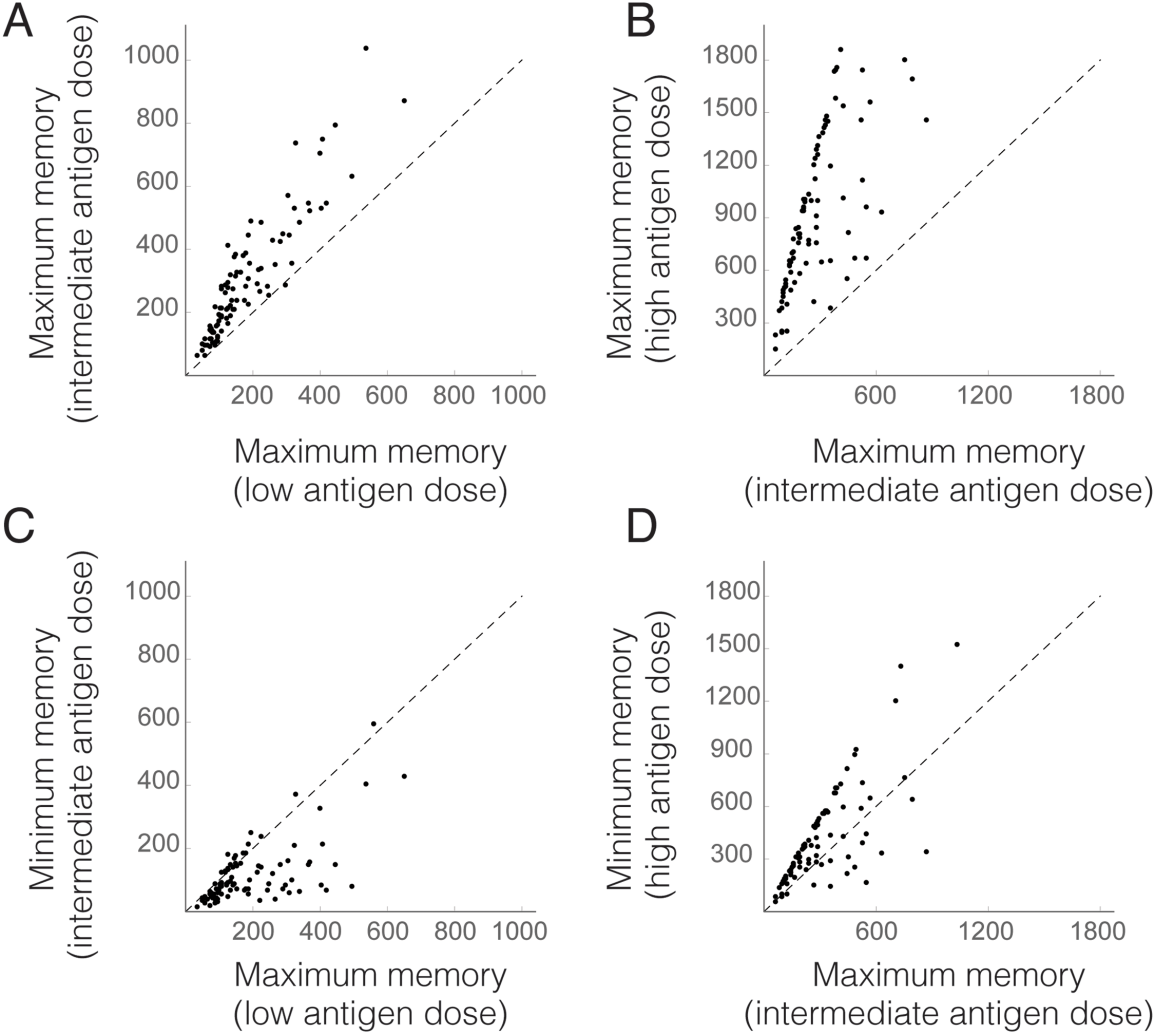
Comparison of maximum and minimum number of memory T cells formed in PB vaccines using different total antigen doses. Performance of PB protocols defined as described in Fig 4 for low, intermediate and high vaccine doses (the rest of parameter values are randomly chosen as in Fig 4). A) Each dot represents the maximum number of memory T cells for PB protocols with low and intermediate antigen doses. All the dots are above the line of equation *x* = *y* (dashed line), which implies that it is possible to produce more memory T cells with intermediate than with low antigen doses. B) Same as in A for intermediate vs high antigen doses. C,D) The maximum number of memory T cells produced with lower antigen doses can be larger than the minimum of memory T cells created when higher antigen doses are used.

Analyzing the effect of total antigen load on memory formation suggests that increasing antigen doses can raise the number of memory T cells produced by a vaccine. In other terms, for a given total antigen dose it is possible to find a PB regime (i.e., a distribution of such antigen dose between prime and boost inoculations and a particular boost timing) that results in higher memory formation than any PB protocol using lower antigen doses (Fig 5A and 5B). However, it is interesting to remark that PB vaccines can occasionally outperform suboptimal regimes involving higher total antigen doses (see Fig 5C and 5D). Therefore, high vaccine doses do not necessarily entail greater memory production.

### Experimental measure of the effect of boost time on memory T cell formation

According to our model, combinations of prime and boost doses and boost times that maximize the formation of memory T cells vary depending on the amount of antigen delivered with the vaccine. Such dose-dependent effect can be roughly described as follows: for lower antigen doses boosting during clonal contraction increases memory formation, while for higher antigen doses using single inoculation vaccines or high prime doses would be better. Importantly, this effect does not emerge for narrow combinations of parameters, but appears to be consistent across the parameter space. In consequence, it can be understood as a result of the model that does not depend on any particular parameterization thereof.

**Fig 6:**
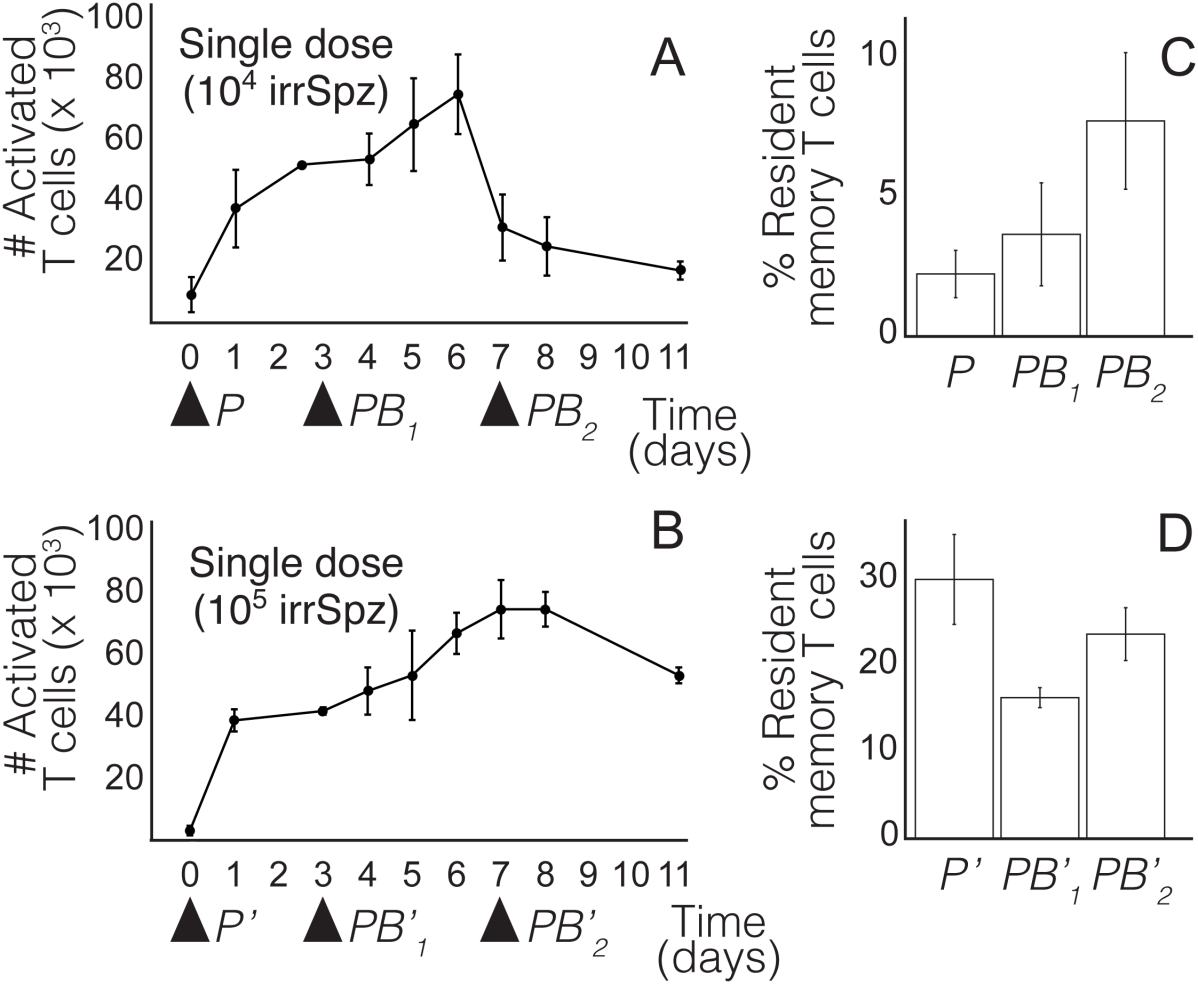
Characterization of clonal expansion and contraction of CD8+ T cells (%CD11a T cells) after immunization with irradiated sporozoites (irrSpz). A) Characterization of clonal expansion and contraction elicited by a single inoculation of 10.000 irrSpz and B) 100.000 irrSpz. C) Comparison of T cell memory formation by protocols *P*, *PB*_1_ and *PB*_2_. *P*: Single inoculation of 50.000 irrSpz; *PB*_1_ and *PB*_2_: prime with 10.000 irrSpz + boost with 40.000 irrSpz three and seven days after prime respectively. D) Formation of memory T cells by protocols *P*′, $PB_{1}^{\prime }$ and $PB_{2}^{\prime }$. *P*′: single inoculation of 140.000 irrSpz; $PB_{1}^{\prime }$ and $PB_{2}^{\prime }$: prime with 100.000 irrSpz and boost with 40.000 irrSpz three and seven days after prime respectively. (*n* = 3 in A, B and D. *n* = 5 in C, *n* being the number of mice used in each experiment). The number of memory T cells is expressed as the percentage of resident memory T cells in the total population of CD8+ T cells. Resident memory T cells where measured 21 days after boosting. Experimental data shown in this picture are provided in Tables A-D in S1 File.

It follows from this result that boost timing should not be defined in terms of a given time interval after prime. Instead, it should be selected relative to clonal expansion and contraction triggered by prime. In order to justify this assertion we have performed a series of experiments to compare the performance of PB regimes in which the same amount of boost is inoculated at fixed times after prime. These regimes consist in priming with two different doses of irradiated sporozoites (10^4^ and 10^5^ respectively) and boosting with 4 × 10^4^ irradiated sporozoites three or seven days later. The performance of these protocols (measured in number of liver-resident memory T cells formed) will be compared to vaccinating with one dose of 5 × 10^4^ and 1.4 × 10^4^ irradiated sporozoites respectively. We remark that days three and seven correspond to clonal expansion and clonal contraction respectively in the first scenario, while they coincide with clonal expansion in the second (see Fig 6A and 6B).

Our results show that relative performance of PB regimes described above is determined by prime dose. In particular, for a prime dose of 10^4^ irradiated sporozoites maximum memory formation is achieved by boosting during clonal contraction. In contrast, if prime dose increases then the differences between the different treatments are smaller, and the relative performance of single inoculation increases (see Fig 6). We remark that our results are consistent within a wide range of model parameters (see caption of Fig 4). The outcome of priming with 10^4^ irradiated sporozoites is similar to numerical simulations corresponding to low or intermediate antigen doses (Fig 6C). Consistently with this observation, the results of increasing prime dose to 10^5^ irradiated sporozoites resemble numerical simulations obtained with higher antigen doses (Fig 6D).

## Discussion

A challenging issue in the design of optimal PB protocols arises from the existence of a wide range of potential strategies to be tested. For instance, a variety of vaccine vectors carrying a given target antigen can be combined in different doses, following different pathways of inoculation, both as prime and boost vehicles [23]. Furthermore, boost agents can be administered (one or several times) at any moment after priming, thus multiplying the number of potential vaccination protocols targeting a particular pathogen [71–73].

The combinatorial nature of the problem of protocol design makes it difficult to assess the performance of alternative vaccine regimes [25]. For instance, an exhaustive experimental procedure to evaluate all the possible protocols that emerge by using four prime agents, combined with four boost agents at four different doses, and five different boosting times would require to test 320 possibilities. Additional considerations such as the choice of target antigens or the via of inoculation raise even more the number of solutions to be tested, further increasing the complication to design effective strategies. Empirical studies consider vaccine doses ranging from 10^2^ to over 10^5^ irradiated sporozoites, and boost timings that vary from a few days to several weeks [62, 74, 75]. Owing to obvious constraints imposed by experimental costs, only a few combinations within such broad ranges are usually tested and compared.

In this work we suggest that taking into account the dynamics of T cell populations can help reducing the experimental effort required to validate and improve PB regimes. Specifically, modeling T cell dynamics allows to understand complex interactions between antigen doses and boost timing, aspects that have been recognized as key factors in the performance of PB vaccines [76, 77]. According to our model, optimal choices of these factors are determined by total antigen load contained in the vaccine (see Fig 4). For a given dose, different patterns of priming and boosting can lead to very heterogeneous results regarding memory formation (Fig 3). Importantly, higher antigen doses do not necessarily translate into greater memory formation, since optimal PB protocols using low antigen dose can result in greater memory formation than suboptimal high-dose regimes (Fig 5).

These results might have practical consequences in the design of optimal PB vaccines. Although irradiated sporozoites have been proven to be powerful immunogens, they raise a series of issues related to their production, cryopreservation and subsequent administration [78, 79]. For this reason, using vaccines with high doses of irradiated sporozoites has long been considered an impractical strategy for human immunization [80]. Therefore, a compromise arises between the amount of irradiated sporozoites administered in a vaccine and the degree of protection it provides. In this context, our results suggest a procedure to distribute a given antigen load between prime and boost inoculations in order to maximize memory production while using lower amounts of irradiated sporozoites.

Furthermore, our model shows that defining boosting times in terms of clonal expansion and contraction triggered by priming allows to compare results from different studies (see e.g. Fig 4). In contrast, lack of references to the status of T cell populations at the moment of boosting makes it difficult to explain observed similarities and divergences between alternative PB strategies in different experimental settings. It also limits the eventual utility of data published in the context of isolated works. For this reason, we suggest that vaccine studies should include the characterization of clonal expansion and contraction triggered by priming (see Fig 6A and 6B). We remark that experimental results shown in this work should be taken as a proof of concept: they show that such characterization of clonal expansion and contraction is feasible, which allows to refer the timing of boosting to the phase of the T cell response elicited by priming. This would allow to build a catalog of T cell responses elicited by different doses of alternative prime agents. In turn, such catalog would provide a useful framework in which to compare the performance of different boost strategies.

The performance of a vaccine in terms of immune protection achieved is a multifactorial problem [72, 81] However, maximizing the production of T cell memory has been suggested as one of the goals of PB vaccines [82]. Our model is focused on analyzing how this goal is related to T cell population dynamics. In consequence, any differences in the performance of alternative strategies can be unequivocally attributed to T cell dynamics alone. In fact, the results presented here do not depend on the particular nature of vaccine vehicles, or the via of inoculation. Therefore, theoretical predictions of the model might be generalized to other cellular-immunity based vaccines. Finally, it is worth noting that even if results shown in this work refer to murine models, similarities in T cell response between mice and humans suggest that our approach might be useful in the development of future human malaria vaccines.

In the current state of knowledge modeling cannot substitute empirical studies in the field of vaccine design. However, we believe that this work can provide a valuable guide to design further experiments needed to improve the efficacy of PB vaccination strategies.

## Material and methods

### Parasites and immunization

Female *Anopheles stephensi* mosquitoes infected with *Plasmodium yoelii* 17 × NL strain were purchased from the New York University insectary. *P. yoelii* sporozoites were isolated from the salivary glands of infected *A. stephensi* mosquitoes 14 days after receiving an infectious blood meal. Sporozoites for immunization were attenuated after giving 15,000 rads by a gamma-irradiator. Mice were immunized with 10^4^, 4 × 10^4^ or 10^5^ irradiated sporozoites suspended in RPMI with 2% mouse sera by intravenous inoculations.

### Ethics statement

All mice were maintained under standard conditions in The Laboratory Animal Research Center of The Rockefeller University. All animal experiments were carried out in strict accordance with the Policy on Humane Care and Use of Laboratory Animals of the United States Public Health Service. The protocol was approved by the Institutional Animal Care and Use Committee (IACUC) at The Rockefeller University (Assurance # A3081-01). Mice were euthanized using CO2, and every effort was made to minimize suffering.

### Antibodies

The following monoclonal antibodies (mAb) were purchased from BioLegend (San Diego, CA) and used for a flow cytometric analysis: purified anti-CD8 (clone 93), Alexa Fluor 647, anti-CD3, anti-CD1a, CD62L, CD44, CD127 and anti-CD16/CD32 FITC-labeled anti-CD8 (clone 53-6.7), PE-Cy7-labeled anti-CD3 (clone 17A2), PerCP-Cy5.5-labeled anti-CD62L (clone MEL-14), PE-labeled anti-CD11a (clone 2D7) and APC-Cy7-labeled anti-CD44 (clone IM7).

### Flow cytometric analysis

Murine cells were incubated with unlabeled anti-CD16/CD32 mAb for 10 min at room temperature and later incubated with the respective mAbs described in the preceding section. Flow cytometric data collection was performed using an LSR II Flow Cytometer (BD Biosciences, San Jose, CA). Subsequent data analyses were performed using FlowJo software (Tree Star Inc.). For the staining of circulating CD8+T cells, the blood was taken from tail vein puncture with minimal restraint. Resident memory CD8 T cells: CD44highCD62L- CD127+. Activated T cells (Effector +effector memory CD8 T cells): CD44+CD62L-CD127-CD11a+.

### Blood samples and purification of lymphocytes from livers

Blood samples were gathered specifically for this study. In order to do that we perfused the mice from the left ventricle with 10ml of PBS1X after cutting the portal vein. Livers were removed from the mice, smashed, and filtered using a strainer of 70 *μ*m. We diluted the pellet with 40% Percoll (50 millions/ 10 ml) containing 100 *μ*/ml of heparin and then loaded on a layer of (20ml) 70% of Percoll solution followed by centrifugation at 2000 RPM for 20 min at R.T with the brakes turned off. We aspirated the cells from the Percoll interface and harvested by centrifugation and washed twice with PBS1X 5% FBS.

### Computational methods

Numerical simulations of the models presented here have been performed in Wolfram Mathematica 10.0.

## Supporting information

S1 File Brief outline of the main assumptions of the models used in this article and their hybrid automaton representation. A series of four tables is included with the raw experimental data shown in Fig 6.
(PDF)
