# Supplementary material for: Modeling the effect of boost timing in murine irradiated sporozoite prime-boost vaccines
Source: PLoS One. 2018 Jan 12;13(1):e0190940. doi: 10.1371/journal.pone.0190940 (PMC5766151; doi:10.1371/journal.pone.0190940)
Supplement: S1 File — A series of four tables is included with the raw experimental data shown in Fig 6. (PDF) [file pone.0190940.s001.pdf]

Supplementary Information for: Modeling the effect of boost timing in prime-boost vaccines against malaria

Cristina Fernandez-Arias[1], Clemente F. Arias[2,3,*], Min Zhang[4], Miguel A. Herrero[3], Francisco J. Acosta[5], and Moriya Tsuji[1]

[1] HIV and Malaria Vaccine Program, Aaron Diamond AIDS Research Center, Affiliate of The Rockefeller University, New York, NY, USA
[2] Grupo Interdisciplinar de Sistemas Complejos (GISC), Madrid, Spain
[3] Departamento de Matemática Aplicada. Universidad Complutense de Madrid, Madrid, Spain
[4] Department of Pathology, University of New York, NY, USA
[5] Departamento de Ecología. Universidad Complutense de Madrid, Madrid, Spain

*Corresponding author: tifar@ucm.es

A. Outline of population mechanics

Population mechanics is based on the following assumptions:

1. T cell populations are elastic and show inertia. This statement is motivated by the dynamics of effector T cells during immune response to acute infections, specifically by the fact that T cells continue to proliferate even after the pathogen has been effectively neutralized (see figure below).

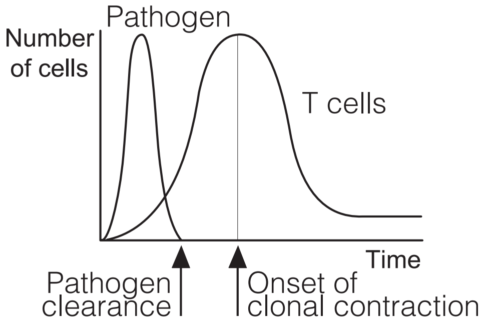

Inertia and elasticity draw clear analogies with models of Classical Mechanics, and call for the use of second order differential equations at the modeling level. From this viewpoint, the dynamics of a cell population results from the balance between opposite forces that tend to increase or decrease the number of cells. In particular, if a cell population x is subject to m different forces $F_1, \cdots, F_m$, then:

$$x''(t) = F_1 + \cdots + F_m$$

2. Proportional distribution of forces. If n T cell populations compete for a given force $F(t)$, then the magnitude of the force perceived by population i is given by:

$$F_i(t) = \frac{x_i(t)}{x_1(t) + \cdots + x_n(t)} F(t), \text{ for } i = 1, \cdots, n.$$

where $x_i(t)$ is the number of cells of population i at time t.

Models of population mechanics Based on the previous assumptions, it is possible to model the dynamics of multiple cell populations that compete for different forces. For instance, let x_1, x_2 and x_3 three cell populations, and suppose that x_1 and x_2 compete for a given factor F_a, while a second factor F_b is shared between populations x_1 and x_3. The corresponding mathematical model takes the following form:

$$\begin{cases} x_1(t)'' = -c_1 x_1'(t) - k_1 x_1 + \dfrac{x_1(t)}{x_1(t)+x_2(t)}\lambda_{1,a}F_a(t) + \dfrac{x_1(t)}{x_1(t)+x_3(t)}\lambda_{1,b}F_b(t) \\ x_2(t)'' = -c_2 x_2'(t) - k_2 x_2 + \dfrac{x_2(t)}{x_1(t)+x_2(t)}\lambda_{2,a}F_a(t) \\ x_3(t)'' = -c_3 x_3'(t) - k_2 x_3 + \dfrac{x_3(t)}{x_1(t)+x_3(t)}\lambda_{3,b}F_b(t) \\ F_a'(t) = f_a(t; F_a, x_1, x_2) \\ F_b'(t) = f_b(t; F_b, x_1, x_3) \end{cases}$$

Parameters c_i and k_i represent the damping and elastic coefficients of population i. Parameter $\lambda_{i,k}$ describes the intensity of the force perceived by population i per unit of factor F_k. Finally, the dynamics of factors F_a and F_b is given by certain functions f_a and f_b, which in turn may depend on the size of the cell populations.

Population mechanics models are of a modular nature and can be easily pieced together to account for additional populations. For instance, adding a fourth population that competes for factor F_a in the previous example yields:

$$\begin{cases} x_1(t)'' = -c_1 x_1'(t) - k_1 x_1 + \dfrac{x_1(t)}{x_1(t)+x_2(t)+x_4(t)}\lambda_{1,a}F_a(t) + \dfrac{x_1(t)}{x_1(t)+x_3(t)}\lambda_{1,b}F_b(t) \\ x_2(t)'' = -c_2 x_2'(t) - k_2 x_2 + \dfrac{x_2(t)}{x_1(t)+x_2(t)+x_4(t)}\lambda_{2,a}F_a(t) \\ x_3(t)'' = -c_3 x_3'(t) - k_2 x_3 + \dfrac{x_3(t)}{x_1(t)+x_3(t)}\lambda_{3,b}F_b(t) \\ x_4(t)'' = -c_4 x_4'(t) - k_4 x_4 + \dfrac{x_4(t)}{x_1(t)+x_2(t)+x_4(t)}\lambda_{2,a}F_a(t) \\ F_a'(t) = f_a(t; F_a, x_1, x_2, x_4) \\ F_b'(t) = f_b(t; F_b, x_1, x_3) \end{cases}$$

We remark that solutions of the previous equations can eventually lead to negative values for some variables. Since negative values are meaningless in our current context, these models must be understood as hybrid dynamical systems. Such systems are characterized by alternating between a collection of discrete states, with transitions from a given state to another being governed by differential equations (see for instance Fig 1 in this file). In case a population reaches value zero, it is reset to zero and removed from the simulations. For more details about hybrid automata see:

- Johansson, K. H. and Lygeros, J. and Zhang, J. and Sastry, S. (2000) Hybrid automata: A formal paradigm for heterogeneous modeling. *Proceedings of the 2000 Ieee International Symposium on Computer-Aided Control System Design*

- Schaft A. J. van der and Schumacher J. M. (2000) *An introduction to hybrid dynamical systems*. Springer. New York, London.

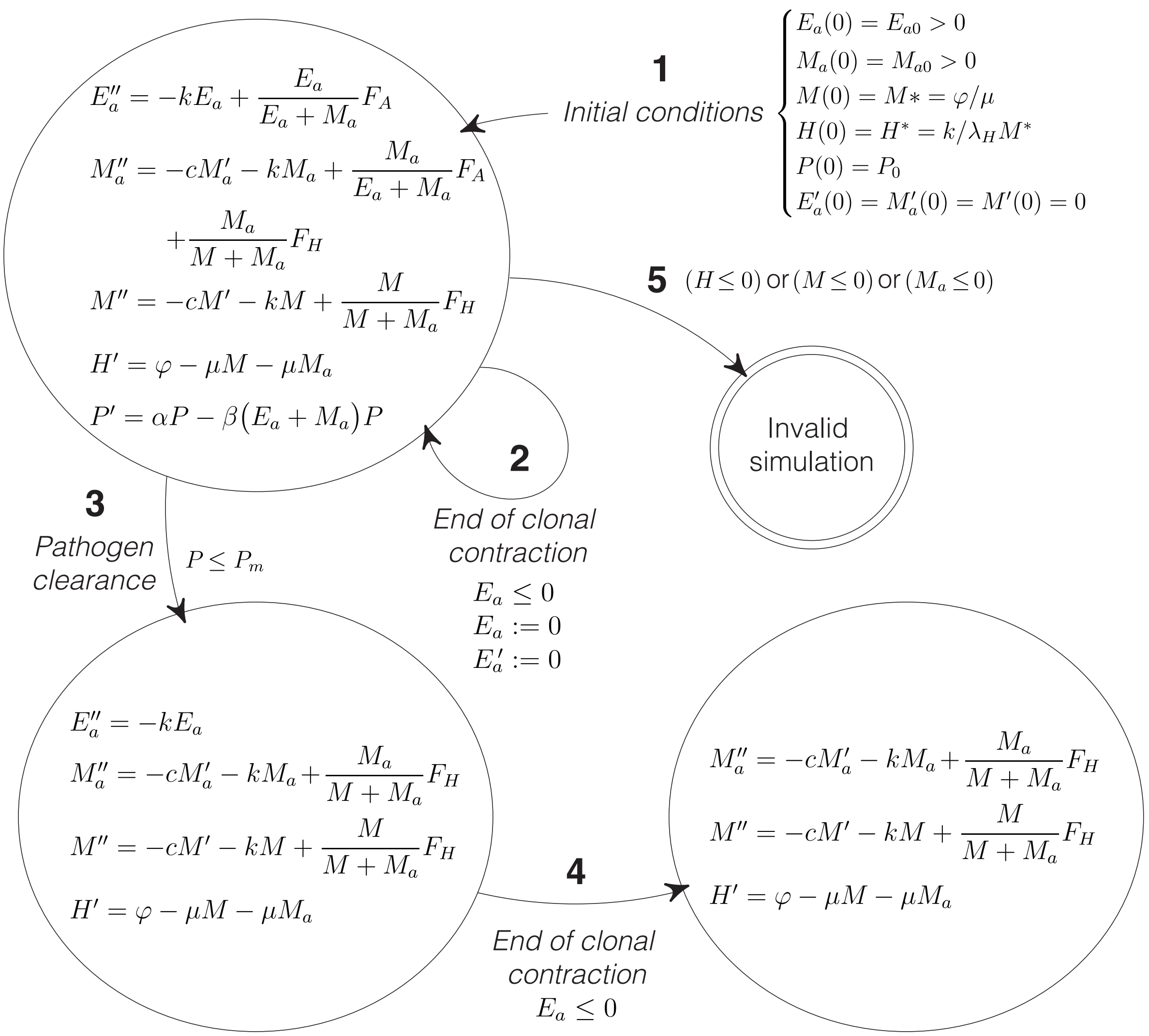

Figure A: **Hybrid automaton representation of equations 1**. Equations 1 describe the situation of effector and memory T cells after activation. (1) Before an immune response both the memory pool M and interleukin H are at equilibrium. This equilibrium corresponds to the values $M = M^* = \varphi/\mu$ and $H = H^* = k/\lambda_H M^*$ (see reference [37] in the main text for further details). The population of effector T cells expands owing to the antigenic force provided by the pathogen. (2) If effector T cells disappear before the end of the infection they are no longer considered in equations 1. (3) On the other hand, if the pathogen falls below a given threshold $P_m > 0$ the infection is controlled, and hence the antigenic force disappears. When this occurs the only force acting on effector T cells is the intrinsic elastic force, while memory T cells continue to compete for the homeostatic force F_H. (4) As consequence of the elastic force, effector T cells eventually disappear, and memory T cells eventually reach a steady state. (5) We will not consider situations in which the homeostatic interleukin or memory T cell populations go to zero. In any of these cases, the chosen set of parameters does not lead to a meaningful simulation.

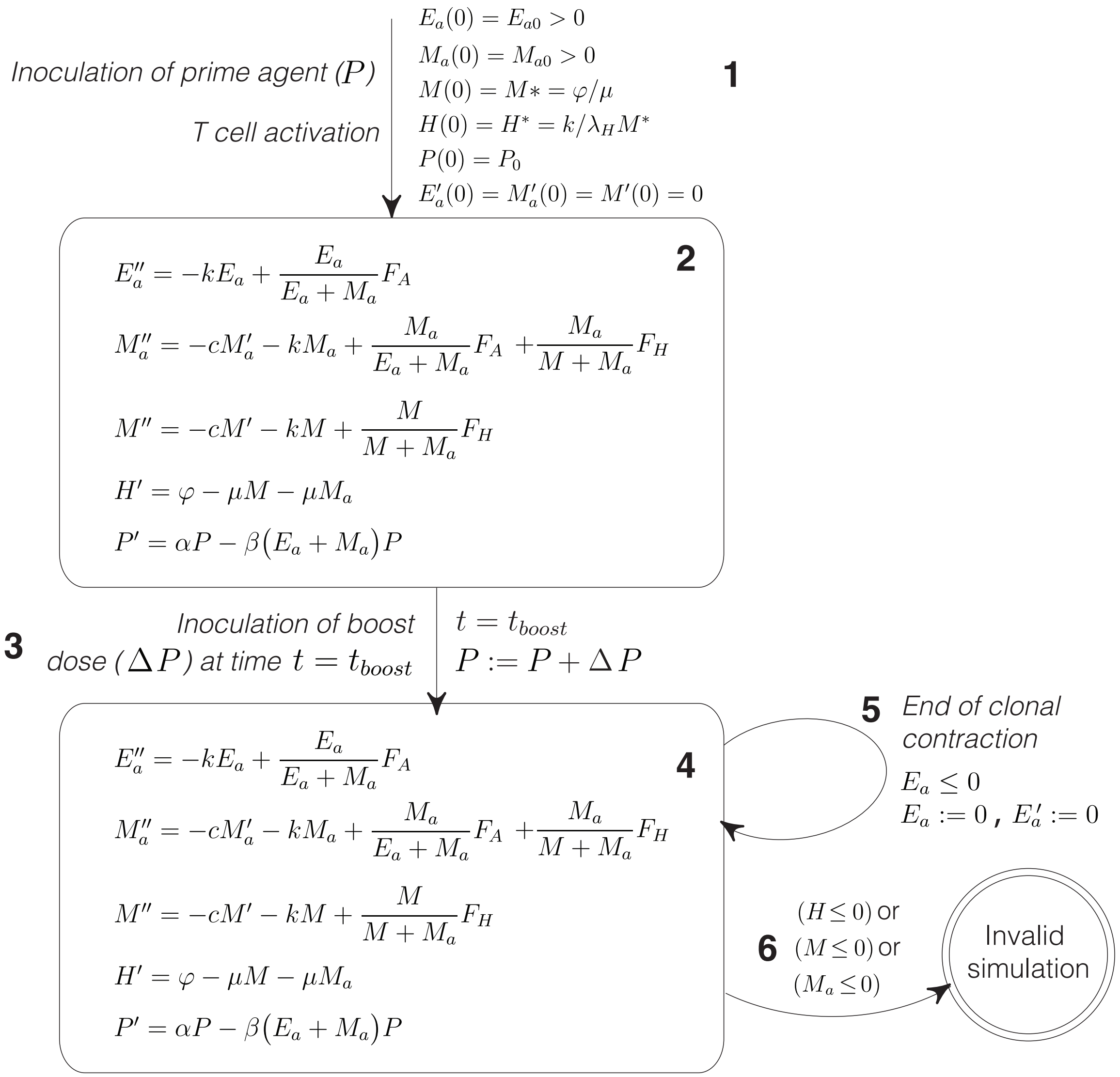

Figure B: **Hybrid automata diagram of the model of homologous PB vaccines.** Notation is consistent with the rest of the models presented in this work.

SM2. Experimental data

Characterization of clonal expansion and contraction

Table A: Number of effector T cells (%CD11ahi) after the inoculation of 10^4 irradiated sporozoites (see Figure 6.A in the main text).

Day	Mouse 1	Mouse 2	Mouse 3	Mean	StDev
0	15	8	4	9	5.568
1	33	50	26	36.3	12.342
2.5	50	51	50	50.3	0.577
4	61	45	50	52	8.185
5	46	72	71	63	14.468
6	68	63	87	72.6	12.663
7	30	41	20	30.3	10.504
8	20	35	18	24.3	9.291
11	15	20	15	16.6	2.887

Table B: Number of effector T cells (%CD11ahi) after the inoculation of 10^5 irradiated sporozoites (see Figure 6.B in the main text).

Day	Mouse 1	Mouse 2	Mouse 3	Mean	StDev
0	2	5	3	3.3	1.527
1	35	38	42	38.3	3.512
3	42	40	42	41.3	1.155
4	40	55	48	47.6	7.505
5	40	50	68	52.6	1.189
6	60	73	65	66	6.557
7	63	80	78	73.6	9.291
8	70	71	80	73.6	5.507
11	55	50	53	52.6	2.516

Formation of memory T cells

Table C: Memory T cell (% of Resident Memory T cells) formed in three vaccination protocols (P: single inoculation of 10^4 irradiated sporozoites, PB_1: priming with 10^4 and boosting three days later with 4×10^4 irradiated sporozoites, and PB_2: priming with 10^4 and boosting seven days later with 4×10^4 irradiated sporozoites). These data are shown in Figure 6.C in the main text.

Treatment	Mouse 1	Mouse 2	Mouse 3	Mouse 4	Mouse 5	Mean	StDev
P	3	2	3	2	1	2.2	0.837
Treatment	Mouse 6	Mouse 7	Mouse 8	Mouse 9	Mouse 10	Mean	StDev
PB_1	3	4	1	4	6	3.6	1.817
Treatment	Mouse 11	Mouse 12	Mouse 13	Mouse 14	Mouse 15	Mean	StDev
PB_2	7	9	5	6	11	7.6	2.41

Table D: Memory T cell (% of Resident Memory T cells) formed in three vaccination protocols (P: single inoculation of 10^5 irradiated sporozoites, PB_1: priming with 10^5 and boosting three days later with 4×10^4 irradiated sporozoites, and PB_2: priming with 10^5 and boosting seven days later with 4×10^4 irradiated sporozoites). These data are shown in Figure 6.D in the main text.

Treatment	Mouse 1	Mouse 2	Mouse 3	Mean	StDev
P	27	27	36	30.0	5.196
Treatment	Mouse 4	Mouse 5	Mouse 6	Mean	StDev
PB_1	17	17	15	16.33	1.155
Treatment	Mouse 7	Mouse 8	Mouse 9	Mean	StDev
PB_2	23	27	21	23.67	3.055
